# Childhood adversity, mental ill-health and aggressive behavior in an African orphanage: Changes in response to trauma-focused therapy and the implementation of a new instructional system

**DOI:** 10.1186/1753-2000-5-29

**Published:** 2011-09-25

**Authors:** Katharin Hermenau, Tobias Hecker, Martina Ruf, Elisabeth Schauer, Thomas Elbert, Maggie Schauer

**Affiliations:** 1 Department of Psychology, University of Konstanz, Box 23/25, 78457 Konstanz, Germany; 2 Vivo international, Eremo delle Grotte, Ancona, Italy

**Keywords:** violence, aggression, PTSD, mental health, orphans, Tanzania, KIDNET

## Abstract

**Background:**

The number of orphans in Sub-Saharan Africa is constantly rising. While it is known that family or community care is preferable over institutional care of African orphans, little is known about the quality of care in orphanages and possibilities of improvement.

**Study 1:**

**Methods:**

Exposure to traumatic stress, experiences of violence in the home, school and orphanage, as well as mental ill-health and aggression of 38 children (mean age of *M *= 8.64 years) living in an orphanage in rural Tanzania were assessed at two time points. The severity of post-traumatic stress disorder symptoms (PTSD), depressive symptoms, and internalizing and externalizing problems were used as indicators of mental ill-health.

**Results:**

Violence experienced in the orphanage correlated more strongly with all indicators of mental ill-health than violence in the former home, school or neighborhood at time point 1. Additionally, violence experienced in the orphanage had a positive relationship with the aggressive behavior of the children at time point 2.

**Study 2:**

**Methods:**

With the help of the pre-post assessment of Study 1, the implementation of a new instructional system and psychotherapeutic treatment (KIDNET) for trauma-related illness were evaluated.

**Results:**

In response to both, a change in the instructional system and psychotherapeutic treatment of PTSD, a massive decline in experienced violence and in the severity of PTSD-symptoms was found, whereas depressive symptoms and internalizing and externalizing problems exhibited little change.

**Conclusions:**

These studies show that violence, especially in the orphanage, can severely contribute to mental ill-health in orphans and that mental health can be improved by implementing a new instructional system and psychotherapeutic treatment in an orphanage. Moreover, the results indicate that the experience of violence in an orphanage also plays a crucial role in aggressive behavior of the orphans.

## Background

In Sub-Saharan Africa the consequences of poverty and the AIDS pandemic have led to constantly rising numbers of orphans and vulnerable children (OVC), as is the case for Tanzania and its 2.6 million orphans as of 2008 [1]. These children live either in extended families, foster families, orphanages, or just on the streets [2,3]. While there has been some research on community care [4,5], little is known about conditions in African orphanages. Some studies from different countries suggest important factors determining the well-being of children in orphanages, such as a secure bonding with a caregiver or living in family-like groups [6-9]. Secure attachment is hindered if caregivers extensively employ adverse conditions including violence in parenting. However, there has been no research to date on the interrelation between violence and mental ill-health in children living in orphanages.

Traditionally, OVC stay with extended family. But due to rising numbers of OVC, families' resources are overstrained [10,11]. As a consequence, most experts argue in favor of supporting families through community-based care and focus on the evaluation of these programs [5,12]. Furthermore, it is known that institutional care may lead to detrimental effects concerning the child's development [13]. Although many orphanages exist and care for OVC, a detailed evaluation of education and care in orphanages lacks in most cases. However, some studies have examined aspects of how orphanages could be improved [6,8]. For example, Wolff, Dawit and Zere [14] restructured an orphanage in Eritrea overtly in order to improve the well-being of its resident children. A stable bond with a caregiver and a particular approach of caretaking seemed to be especially important [9]. Studies from other countries like India and Russia support these findings [6,7]. It is obvious that a caregiver's violent behavior could endanger the development of a predictable, emotionally safe connection. Additionally, OVC often experienced violence and neglect in their family of origin and in neighborhood or school [15]. Corporal punishment is still used worldwide in homes and schools [16,17], although studies show that corporal punishment is linked to mental ill-health and aggression in children [16,18,19]. Corporal punishment is not explicitly prohibited at home and school in Tanzania [20]. To date no prevalence rates for Tanzania are available [20], but Straus [17] reported that more than two thirds of Tanzanian students did not strongly disagree that they were frequently spanked or hit before the age of 12 years. In comparison with students from other countries, Tanzanian students reported the second highest percentage.

It has been repeatedly shown that experiences of violence or neglect in childhood often lead to mental ill-health, like post-traumatic stress disorder (PTSD) or depression [21-24]. Due to their living conditions, OVC are often exposed to several traumatic stressors. According to the building block effect, repeated traumatic experiences culminate into a higher risk for PTSD [25]. Moreover, abuse and neglect can lead to aggressive behavior in the children themselves [26,27]. Without secure attachment a child might have problems developing strategies of self-regulation [28,29]. Therefore, it is important to know which adverse conditions, and violent punishment in particular, may have the biggest impact on mental health of children, who are living in orphanages, and how types of care affect healthy development, mental well-being and a child's preparedness for aggressive behavior.

The first study examined the relations of exposure to violence and mental ill-health in an orphanage in Tanzania. It was hypothesized that violence experienced in the family of origin, the school, neighborhood, or in the orphanage relates positively to the mental ill-health of the orphans. Additionally, the children's aggressive behavior was examined. A positive relationship between exposure to violent acts and aggressive behavior in the children was expected. The second study dealt with the evaluation of an intervention in the same orphanage. To improve the living conditions of the children a new instructional system was implemented that placed a ban on any violent punishment by caregivers and introduced positive parenting strategies. Furthermore, all children with a PTSD, diagnosed according to DSM IV criteria, received KIDNET, [30] a child-friendly version of narrative exposure therapy (NET) [31]. A time period of six months allowed the caretakers to get used to the new strategies and the children to profit from the changes, but also to recover from PTSD. A decline in reported violence in the orphanage as well as in mental ill-health was expected six months later.

## Study 1

### Methods

#### Participants

The examined children live in a non-governmental orphanage in the Southern Highlands of Tanzania, situated near a small village in a rural area. The orphanage consists of four houses with nine to twelve children of different ages and sexes with two caretakers for each house. The caretakers had mostly no preparatory qualification for their jobs as caretakers and only primary school education. Children were either full or partial orphans or had been severely abused or neglected by their families and were therefore taken into orphan care. Children, who were seven years or older, were interviewed for two hours on average at time point 1 (t1) and six months later at time point 2 (t2). The younger children could only answer part of the questions. Further qualitative information concerning mental ill-health, especially of the younger children, was gained through behavioral observation by the investigators who lived five weeks (during t1) and three weeks (during t2) with the children. In general, the analyses included all children (*N *= 38; 53% boys) who were in the orphanage during both assessment periods. The mean age was *M *= 8.64 years (range 3 - 16) at t1 and *M *= 9.16 years (range 3 - 16) at t2. The Tanzanian and German board of the organization managing the orphanage gave their consent and ethical approval.

#### Materials

The interview sets were basically identical for both assessments. All instruments were applied as a structured interview by clinicians with extensive working experience including an East African context. This experience and the application through an interview allowed the interviewers to complete the interview with many children of seven years or older.

Socio-demographic data: The first part of the interview consisted of socio-demographic information, in which the children were also asked about their parents, the reason for death of the parents and about relationship to relatives.

Physical health: The children were interviewed about their physical health in the past four weeks based on a checklist (concerning cough, stomach pain, tuberculosis, headache, malaria, flu, pain, diarrhoea, fever/shivering, skin rush/scabies, and vomiting) [32].

Stressful and traumatic experiences: In the subsequent section of the interview, the children were asked about their experiences of violence. This included physical, psychological and sexual violence as well as neglect and witnessed violence. The children were asked 41 questions about violence (following C. Catani at http://www.vivo.org). At t1 they were asked about the experienced violence at home, in school or neighborhood, and in the orphanage during their whole lifetime. At t2 they were only interviewed about experienced violence in neighborhood or school and the orphanage in the last six months.

Mental health: Concerning the mental health of the children, internalizing and externalizing problems, PTSD, and depression were assessed.

Internalizing and externalizing problems: The self-evaluation of strengths and difficulties was assessed with the Strengths and Difficulties Questionnaire (SDQ) [33]. The SDQ comes with good psychometric properties and is internationally implemented [34]. This study uses the self-report version for children from 11 to 17 years. It consists of 25 statements with the possible responses that the statement is *not true, somewhat true *or *certainly true *for themselves. Each of the five subscales (conduct problems, hyperactivity, emotional symptoms, peer problems and prosocial behavior) consists of five items. The total difficulties score is generated by summing the scores of all items, except the items for prosocial behavior, and ranges from 0 to 40. A score over 20 indicates an abnormal amount of internalizing and externalizing problems. The total difficulties score is a good measure for a general impression of internalizing and externalizing problems and is, therefore, a sufficient measure for this study.

Post-traumatic stress disorder: The UCLA PTSD Index for Children DSM IV [35] was used to screen for exposure to traumatic events and for symptoms of PTSD. This instrument was originally constructed as a self-report and assesses the severity of symptoms based on the frequency of symptoms reported by the child. The occurrence of each DSM-IV symptom within the last month is scored on a scale ranging from *none of the time *to *most of the time*. Thus, an overall PTSD severity score can be calculated by summing the scores for each question, which results in a maximum possible score of 68. The UCLA PTSD Index shows good psychometric properties and has been successfully utilized in non-western settings [21,23].

Depression and suicidality: Depression and suicidality were assessed with the Mini-International Neuropsychiatric Interview kid for children and adolescents (M.I.N.I.; Section A and C) [36]. Additionally, the severity of depressive symptoms was assessed by means of the Children's Depression Inventory (CDI) [37]. The CDI is a reliable and well-tested clinical research instrument designed for school-aged children and adolescents. It has been successfully implemented in Tanzanian settings [3,38]. Originally it is administered as a self-report instrument and evaluates the severity of specific depressive symptoms. It contains 27 items with three statements each and the child has to choose which statement fits best. For each item, the points range from 0 to 2, where higher values represent more clinically severe symptoms. Thus, the possible maximum score is 54.

Aggression: Aggressive behavior was assessed at t2 with the Reactive-Proactive Questionnaire [39]. The children were asked how often they have exhibited a specific aggressive behavior, in which they have to choose between *never, sometimes *and *often*. One item of originally 23 items was removed, because it was not appropriate for the conditions in rural Tanzania (Item 18: *Made obscene phone calls for fun*) and two items were slightly rephrased for a better understanding (Item 4: *students *replaced with *children *and Item 9: *gang fight *replaced with *fight*). The sum of the points assigned to the answer represents the total aggression and ranges from 0 to 44.

#### Procedure

The first assessment in March 2010 was carried out by four of the authors. They worked together with trained translators and stayed for five weeks in the orphanage. The second assessment was carried out in September 2010, six month after the first assessment, by the two other authors (KH and TH) again with trained, but now different translators. This second team of interviewers was blind with respect to any information gathered during the first assessment and did not know who had received psychotherapeutic assistance. The second assessment was completed after three weeks. The translators were trained before both assessments and the interviewers had standardized the form of assessment by practicing in joint interviews to achieve a high inter-rater reliability. All instruments were translated word-by-word into Kiswahili and the translation was intensely discussed to guarantee a precise translation.

Every child of seven years or older was interviewed alone in a quiet place by one interviewer and one translator. To provide a trustworthy environment, the girls were interviewed by at least one woman. The interview took two hours on average. Children were assured that the whole interview was confidential and that there would be no punishment for whatever information was given. The amount of breaks varied with the child's ability to concentrate. Children received drinking water and a fixed number of sweets during the interview to help them to stay focused. Children were encouraged to draw a picture or to play their favorite game at the end of the interview. In addition, the behavior of all children was observed in their typical daily surrounding. During the periods of assessment, interviewers and translators stayed in the orphanage and shared the meals with the children and played with them in their free time.

#### Analyses

All variables except one met the preconditions for the analyses. The sum of depressive symptoms at t1 was not distributed normally. Therefore, the Spearman coefficient was computed for correlations using the sum of depressive symptoms at t1. The Pearson coefficient was calculated for all other correlations. The Bonferroni correction was used in cases of multiple testing to prevent alpha-inflation. All hypotheses about mental health were subdivided in specific hypotheses for PTSD, depression, and internalizing and externalizing problems. Due to the directional hypotheses, analyses were computed one-tailed. According to the age of the children, n = 22 children could be included in the analyses of the severity of PTSD symptoms, whereas n = 33 children were included concerning the severity of depressive symptoms and internalizing and externalizing problems. The analysis of the relation between experienced violence in the orphanage and aggression included n = 29 children.

### Results

#### Experiences of Violence

At t1 the children reported a mean of *M *= 5.59 (*SD *= 5.42, range 0 - 19) different forms of violence experienced in the family of origin before entering the orphanage. On average they reported to have experienced *M *= 2.30 (*SD *= 1.98, range 0 - 7) different forms of violence in school or neighborhood. Concerning the violence experienced in the orphanage children specified an average of *M *= 4.03 (*SD *= 3.99, range 0 - 17) different forms of violent events. At t2 the children reported that they had experienced on average *M *= 2.57 (*SD *= 1.81, range 0 - 6) different forms of violence in school or neighborhood and *M *= 1.93 (*SD *= 2.40, range 0 - 8) different forms of violence in the orphanage in the past six months.

#### Mental health

At t1 14 children fulfilled the criteria for PTSD, seven of which still fulfilled the diagnosis at t2. Additionally, one child was diagnosed with PTSD at t2 who did not fulfill the criteria at t1. Of the five children, who were diagnosed with a Major Depression episode at t1, only one child fulfilled the criteria for a diagnosis at t2. At t1 six children showed an abnormal amount of internalizing and externalizing problems. The criteria were still fulfilled by five children at t2.

#### Correlations at t1

At t1 a positive relationship between experienced violence and mental ill-health was expected. Within each specific directional hypothesis the correlation with experienced violence in the orphanage, in neighborhood or school, and the home was tested. All analyses were performed with an alpha-level of significance of α = .017 due to the Bonferroni correction within each specific hypothesis. A significant correlation was found between the experienced violence in the orphanage and the severity of PTSD symptoms (*r *= .60, *p *< .01) and between experienced violence in the home and severity of PTSD symptoms (*r *= .50, *p *< .01). However, no significant correlation between experienced violence in neighborhood and school and PTSD symptoms (*r *= .20, *p *> .18) was found.

The relationship between experienced violence and the severity of depressive symptoms was confirmed by a significant correlation between the sum of violence experienced in the orphanage and the severity of depressive symptoms (*r *= .43, *p *< .01). There was no such relationship with violence experienced in school and neighborhood (*r *= .14, *p *= .22) or in the former home (*r *= .37, *p *> .017).

There was a significant correlation between violence experienced in the orphanage and internalizing and externalizing problems (*r *= .61, *p *< .01) as well as between violence experienced in the former home and internalizing and externalizing problems (*r *= .52, *p *< .01). Additionally, a significant correlation between violence experienced in neighborhood or school and internalizing and externalizing problems (*r *= .38, *p *= .015) was found.

#### Aggression

**Figure 1:**
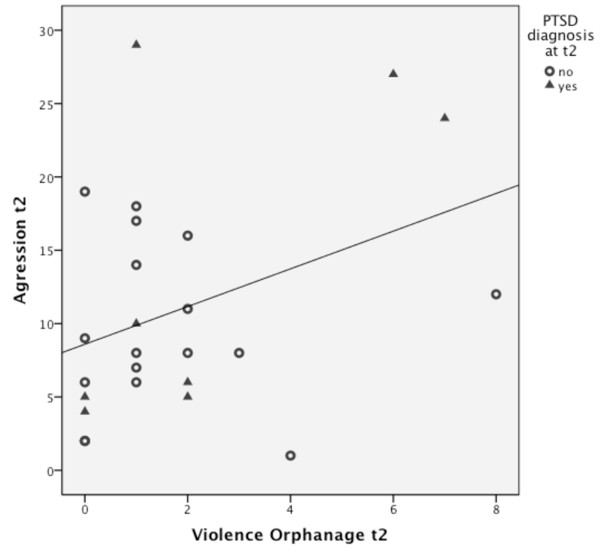
**Scatter plot of the sum of violence experienced in the orphanage and the sum of aggressive behavior at t2**. The line represents the relationship between experienced violence and aggressive behavior at t2.

To test the assumption of a positive correlation between violence experienced in the orphanage and aggressive behavior at t2, the alpha-level was set to α = .05. The analysis showed a significant positive correlation between violence experienced in the orphanage and aggressive behavior at t2 (*r *= .48, *p *< .01). The relationship is shown in Figure 1.

## Study 2

### Methods

#### Participants

Study 2 included the same participants as Study 1. Their characteristics were described above.

#### Materials

For the evaluation of the intervention the same interviews were used as described in Study 1.

#### New Instructional System

The new instructional system included training sessions for the caretakers that aimed for a better understanding towards the children and for a positive relationship between caretaker and child in order to reduce violent punishment and to foster secure bonding.

1. HIV: As many children were orphaned due to HIV/AIDS, caretakers were trained on possible ways of transmission. It turned out that many of them were not at all informed and therefore avoided, for example, skin-to-skin contact with children, whose parents died due to HIV/AIDS. The aim was to reduce prejudices und insecurity of the caretakers in order to support a close relationship to the children.

2. Developmental Stages, Windows of Opportunity, Attachment, and Bonding: Some theoretical knowledge about developmental stages, attachment, and bonding was given to the caretakers to foster their understanding and empathy towards the children.

3. Grief: As many of the children have lost their parents also some knowledge about grief in children was given in theoretical lectures. Again the aim was to foster the understanding of the caretakers for the children's experiences.

4. Positive Parenting Strategies according to the Oregon Model [40] were taught. Giving good directions, establishing clear and age-appropriate expectations and rules, tracking of directions and cooperation, positive reinforcement, effective discipline strategies, and the establishment of a token system had primary focus. Theoretical lectures and practice in role-plays were used to teach the elements of the Oregon Model. Additional handouts were prepared and translated into Kiswahili to ensure retention.

After the workshop a special needs teacher, who graduated at a German college, supervised the implementation of the newly developed instructional system for six months. In addition, any form of physical punishment was banned and all caretakers were informed that any use of physical punishment and other forms of maltreatment, such as punishing children by sleeping on the floor, would lead to instant dismissal. Moreover, all boys and girls of twelve years or older were also informed about this ban and about zero tolerance of violence, also among peers, and received sex education, including information on HIV/AIDS.

#### KIDNET - Narrative Exposure Therapy for Children

The theoretical background and treatment rationale is described in detail elsewhere [30,31,41]. In brief, during KIDNET the child, with the assistance of the therapist, constructs a chronological narrative of his or her whole life with a focus on exposure to traumatic stress. Empathic understanding, active listening, congruency and unconditional positive regard are key components of the therapist's behavior. For traumatic experiences the therapist asks in detail for emotions, cognitions, sensory information and physiological reactions and records these meticulously, linking them to an autobiographical context, namely time and place. In order to meet the needs of children, illustrative and creative elements are employed to pursue the goal of memory reorganization.

#### Procedure

Based on the findings of the first assessment and in cooperation with the administration of the orphanage, a new instructional system was introduced in March 2010 that included non-violent, positive parenting strategies based on reinforcement learning. New strategies to handle difficult situations without violence were trained with the caretakers. During two weeks of training all caretakers of the orphanage were trained in 10 one-hour sessions. In addition, the authors treated only children with PTSD, diagnosed according to DSM IV criteria, with Narrative Exposure Therapy for children (KIDNET) [30,31,41]. Each of these children received 5 to 6 sessions of 90 minutes. While the psychotherapeutic treatment was administered to reduce the symptoms of children diagnosed with PTSD, the instructional changes aimed at providing a good atmosphere to all children and at preventing them from new experiences of violence. As described above, a second assessment was carried out six month after the first assessment in order to evaluate the new instructional system.

#### Analyses

As described for Study 1, the sum of depressive symptoms at t1 was not distributed normally. Thus, the Wilcoxon rank-sum test was computed to compare the two times of measurement of this variable. All other comparisons of t1 and t2 were analyzed by computing *t*-tests for dependent variables. To test the specific hypotheses an alpha-level of α = .05 was used. In cases of directional hypotheses, analyses were computed one-tailed. According to the completeness of datasets for t1 and t2, the analyses of the severity of PTSD symptoms included n = 20 children, whereas the analyses concerning the severity of depressive symptoms included n = 22 children and concerning internalizing and externalizing problems n = 26 children. The analyses of correlations between the severity of PTSD symptoms and different types of experienced violence included n = 25 children.

### Results

#### Differences between t1 and t2

There was a significant drop of violence experienced in the orphanage from *M *= 4.48 (*SD *= 4.14) at t1 to *M *= 1.93 (*SD *= 2.40) at t2 (*t*[28] = 3.42, *p *< .01). Cohen's *d *indicated a large effect (*d *= 0.86).

The assumption of a decline in mental ill-health comparing t1 and t2 was subdivided into specific hypotheses. Between t1 (*M *= 21.95, *SD *= 17.43) and t2 (*M *= 14.65, *SD *= 10.95) a significant decline (*t* [19] = 2.46, *p *= .01) in the severity of PTSD symptoms was found. An average effect was found with Cohen's *d *= 0.50. However, there was no significant decline in the mean severity of depressive symptoms using Wilcoxon rank-sum test (*z *= -0.28, *p *= .78) between t1 (*M *= 7.36, *SD *= 7.54) and t2 (*M *= 6.36, *SD *= 4.16). Comparing the average sum of internalizing and externalizing problems at t1 (*M *= 11.88, *SD *= 5.27) and t2 (*M *= 9.73, *SD *= 7.89) no significant difference was found (*t* [25] = 1.12, *p *= .14). Correspondingly, Cohen's *d *showed a small effect with *d *= 0.32.

#### Correlations at t2

It was assumed that no correlation between violence experienced in the orphanage and mental ill-health at t2 exists. A level of significance of α = .05 was used to test the specific hypothesis for every indicator of mental ill-health. There was no significant correlation between violence experienced in the orphanage and PTSD symptoms (*r *= .23, *p *= .26). Additionally, no significant correlation between violence experienced in the orphanage and depressive symptoms (*r *= .16, *p *= .47) as well as between violence experienced in the orphanage and internalizing and externalizing problems (*r *= .28, *p *= .17) was found.

## Discussion

Sub-Saharan Africa struggles with constantly rising numbers of orphans and vulnerable children [1]. Up until today little has been known about their mental ill-health as consequences of their experiences. Therefore, we interviewed all children in an orphanage before and six months after the implementation of a new instructional system.

All in all, the findings are consistent with the expected relationship between experienced violence and mental ill-health of the children living in the orphanage (Study 1). The correlation with violence experienced in the orphanage is the strongest for all three indicators of mental ill-health at t1. Additionally, correlations with other forms of experienced violence are significant for PTSD symptoms as well as internalizing and externalizing problems at t1. Furthermore, a relationship between experienced violence and aggressive behavior in the children was observed at t2. After the implementation of the new instructional system and individual trauma therapy for all children suffering from PTSD (Study 2), the violence experienced in the orphanage declined, but the expected decline in mental ill-health was statistically significant only for PTSD. As expected, the relationship between violence experienced in the orphanage and mental ill-health could not be found at t2.

The relationship between experienced violence and mental ill-health is concordant with other research on the consequences of violent experiences [22,23]. However, the findings suggest that the violence experienced in the orphanage plays an essential role in the ill-mental health of the children, even more important than the amount of violence experienced in the family of origin, before entering the orphanage, or in school and neighborhood. Therefore, it can be assumed that the parenting style of the caretakers plays a crucial role for the mental health and development of the children. The decline in PTSD severity and violence experienced in the orphanage after the implementation of the new instructional system and the individual trauma treatment indicates a successful change in caretaking strategies. The influence of the new instructional system and the psychotherapeutic treatment of PTSD with KIDNET cannot be separately examined. However, the decline in violence and the non-existing correlation of experienced violence and PTSD severity at t2 argue for an influence not only of KIDNET, but also of the instructional system, as KIDNET has no influence on the use of violence by caretakers and not all children received KIDNET. A decline in depressive symptoms and internalizing and externalizing problems was expected, but not found. The mean severity of these symptoms was already rather low in the first assessment, which may have led to a floor effect. Moreover, the change in depressive symptoms may take more time under these conditions.

Caretaking strategies that avoid violent punishment, but provide possibilities for a secure bonding, can ameliorate the mental health of children who experienced violence in earlier settings [8,9]. The orphanage, as the current place of living, can provide a safe place to recover from the violence experienced in other settings. This view is supported by the decline of violent acts and improvement in mental health after implementing the new instructional system. Caretakers without specific pre-training in childcare and with little formal education could understand and apply positive parenting strategies and a zero-violence policy. Although the evidence for the detrimental effects of exposure to institutional care per se is overwhelming, the aspects of quality matter [6-8]. Furthermore, the relation between experienced violence and aggression is important. However, the data give no information about causality. Even though the experienced violence declined in general, more aggressive children nevertheless reported more violence experienced in the orphanage. Aggressive behavior in children can lead to violent reactions of other children or caretakers, while experienced violence can correspondingly lead back to aggressive behavior. Similar findings were reported from other studies concerning organized and domestic violence [26,27]. Experienced violence and the related aggressive behavior might lead to a climate in the orphanage that upholds mental ill-health and violent behavior of caregivers. This endangers the development of strategies of self-regulation [28,29]. The relationship between experienced violence and aggressive behavior supports the assumption that the violence experienced in the orphanage plays an important role for the mental health of the children.

Some methodological aspects limit the generalization of the findings. Due to the limited number of children, statistical analyses uncovering more complex interactions between multiple variables could not be computed. Information was only gathered from the children's perspective, which holds the risk of a social desirability effect. Although additional information by teachers and caretakers was preferred, caretakers showed big difficulties to provide specific and detailed information about the children. Certainly, representativeness for other orphanages cannot be claimed. However, the consistency with findings from other countries concerning caretaking strategies lends some support to the idea that similar relationships would also be found in other settings. Moreover, important limitations stem from the absence of a control group. Other influences than the implemented intervention, including a change in the instructional system and treatment of PTSD, may have led to a decline in violence as well as to a decline in PTSD symptoms. Therefore, no conclusion about causality can be drawn from the data due to a variety of confounding variables. Likewise, a natural recovery process might be responsible for the decline in PTSD symptoms. However, this process would be fostered by non-violent caretaking. Furthermore, the instruments used were not validated for a Tanzanian population, but they were implemented as structured interviews by clinicians with extensive experience in mental health research in low-income countries and have been successfully tested before in other Sub-Saharan African settings. The translators were extensively trained and the translation was discussed in detail. Nevertheless, cultural bias might have influenced the findings, as questions might not always reflect typical parts of the life of a Tanzanian child.

## Conclusions

Results suggest that violence experienced in orphanages has a bigger impact on children's well-being than violence experienced earlier in the family of origin or when visiting school. These findings support the assumption that, although living in an orphanage increases the risk of mental ill-health in children, a good quality of caretaking can buffer negative effects. Moreover, the study demonstrated a relationship between exposure to violence and aggressive behavior in children, which again supports the assumption that violence experienced in the orphanage has a strong impact on children's development and well-being. The number of orphans and vulnerable children in Sub-Saharan Africa is still growing. If these children have no chance to grow up in good caretaking structures, they may grow into adults with problems of mental ill-health and aggressive behavior. Given the small amount of resources and the short time it took to implement change in this orphanage, this study emphasizes that orphanages in resource poor countries must be supported to implement a structured basic instructional plan, based on principals of primary care attachment, zero-violence and positive parenting.

## Competing interests

The authors declare that they have no competing interests.

## Authors' contributions

KH carried out the second assessment, performed the statistical analyses, and drafted the manuscript. TH carried out the second assessment, performed the statistical analyses and helped to draft the manuscript. MR, ES, TE and MS carried out the first assessment, introduced the instructional system, trained the caretakers, and treated children diagnosed with PTSD with KIDNET. All authors read and approved the final manuscript.
